# Utility of the Basophil Activation Test Using Gly m 4, Gly m 5 and Gly m 6 Molecular Allergens for Characterizing Anaphylactic Reactions to Soy

**DOI:** 10.3389/falgy.2022.908435

**Published:** 2022-05-18

**Authors:** Bertrand Evrard, Justine Cosme, Marion Raveau, Maud Junda, Elodie Michaud, Benjamin Bonnet

**Affiliations:** ^1^Service d'Immunologie, CHU Clermont-Ferrand, Clermont-Ferrand, France; ^2^Laboratoire d'Immunologie, ECREIN, UMR 1019 Unité de Nutrition Humaine, Faculté de Médecine de Clermont-Ferrand, Université Clermont Auvergne, Clermont-Ferrand, France; ^3^Unité d'Allergologie Pédiatrique, CHU Clermont-Ferrand, Clermont-Ferrand, France

**Keywords:** soybean allergy, basophil activation test (BAT), molecular allergen, Gly m 4, Gly m 5, Gly m 6, PR-10, anaphylaxis

## Abstract

There are two major clinically described forms of IgE-dependent soy allergy: (i) a primary dietary form, linked to sensitization against soy storage proteins Gly m 5 and Glym 6, and (ii) a form included in birch-soy syndromes linked to Gly m 4, a PR-10-like allergen. This second form sometimes causes severe systemic reactions, even anaphylaxis, especially on consuming certain forms of soy such as soymilks or smoothies. Skin prick tests and specific IgE assays against soy whole extracts lack sensitivity. Assays of anti-Gly m 4, Gly m 5 and Gly m 6 specific IgEs have been developed to overcome this obstacle, but they unfortunately lack specificity, especially for anti-Gly m 4. We hypothesized that the basophil activation test (BAT) using molecular soy allergens Gly m 4, Gly m 5 and Gly m 6 would both remedy the lack of sensitivity of other tests and offer, through its mechanistic contribution, greater specificity than the assay of anti-Gly m 4 specific IgEs. This would enable the two types of soy allergy to be separately identified. In a characteristic clinical example of PR-10-induced anaphylactic reaction after consuming soymilk, we report preliminary results of Gly m 4-exclusive positivity of BAT supporting our hypothesis. It will be necessary to confirm these results on more patients in subsequent studies, and to specify the place of the BAT in an overall diagnostic strategy. Meanwhile, soy BAT using molecular allergens is a promising diagnostic tool for soy allergy and probably also for follow-up in specific immunotherapies.

## Introduction

Soybean has been consumed in South Asia since ancient times. The plant, *Glycine max*, belongs to the legume family and is widely used for its health and nutritional benefits in both humans and animals (1). Soybean is one of the “big eight” foods responsible for 90% of food allergies (2). However, the prevalence of food allergy to soy remains controversial, and varies greatly from one country to another, ranging in children from 0% in certain countries, such as Greece and Spain, to around 0.4% in the USA (3, 4). Notably, soy is the fifth or sixth most common allergen found in children with atopic dermatitis, and 5–14% of children with an allergy to cow's milk protein develop an allergy to soy when exposed to soy-based formulas (5). More than half of children allergic to soy are cured by age 7, suggesting that this allergy is less prevalent in adults (6).

Soy allergy significantly impacts quality of life because soybean is present in many foods, often discreetly, making eviction difficult. Although soy seems to induce less severe forms of disease than the other priority allergens (7), severe or fatal soy anaphylaxis has been occasionally described in a long timeframe (8).

More than 16 soy allergens have so far been described at molecular level (2). Clinical relevance has not yet been demonstrated for most of them, but three main molecular allergens seem to be of special clinical interest and to correspond to two different forms of the disease. First are two highly abundant storage proteins in soybean seed, Gly m 5 and Gly m 6, respectively from the beta-conglycinin (7S globulin) and glycinin (11S globulin) families (9–11). These allergens, stable to heat and gastric digestion, may be responsible for anaphylactic reactions to all kinds of dietary soybean, including fermented and processed foods. They may be associated with forms of allergy of primary food origin with sensitization through the gastrointestinal tract, in particular linked to cross-allergies to molecular allergens from the same families of storage proteins, especially including those of peanuts (Ara h 1 and Ara h 3) (12, 13). Second is Gly m 4, another medically interesting allergen, which is a PR10-like protein, called starvation-associated message protein (SAM22), with a certain degree of homology with Bet v 1, the major allergen of birch pollen. IgE-dependent cross-reactions between Gly m 4 and Bet v 1 are thus involved in birch-soy syndrome, one of the most important pollen-food syndromes (PFS) to be medically investigated, through a common clinical pattern of severe oral allergy syndrome and anaphylactic reactions (14). Approximately 10% of highly sensitized patients allergic to birch present a cross-allergy to soy and nearly 50% of them have experienced systemic or even anaphylactic reactions (15). The PR-10 family of proteins is heat-sensitive, so these patients will not react to all types of soy foods. Moreover, it is known that the skin prick test (SPT), and the determination of specific IgEs (sIgEs) against whole natural extract of soy, both lack sensitivity when the soy allergy is mediated by the PR-10 family (16). This stems from the difficulty met in extracting the Gly m 4 allergen from whole extracts and the low content of this allergen in them (17).

Component-resolved diagnosis (CRD), based on the use of unit sIgE assays against molecular allergen, Gly m 4 (ImmunoCAP, Thermo Fisher Scientific^®^), can overcome this hurdle. CRD, additionally using Gly m 5 and Gly m 6, is also useful for differentiating between these two types of soy food allergy, whose clinical characteristics differ (9, 16, 18). The results of these tests are always interpreted in the light of medical history, and despite the limitations described above, with the results of the SPT and the determination of sIgE antibodies directed against whole extract of soy (f14).

When the results of molecular sIgE assays are dichotomous (i.e., sIgE against Gly m 5 and/or 6 positive with Gly m 4 sIgE negative, or the reverse), clinical and biological interpretation remains relatively straightforward to determine whether it is a soy allergy initially of food or respiratory origin. However, interpretation of the assays is often hampered by the fact that many patients highly sensitized to birch also have sIgEs that can recognize Gly m 4. For example, in the study by Mittag et al., about 71% of patients with anti-Bet v 1 IgE above 17.5 kU/L also had positive sIgE antibodies to Gly m 4, whereas only 9.6% of them described allergic symptoms after soy food consumption (15). The interpretation of the anti-Gly m 4 sIgE assay thus sometimes requires caution, especially for low values or when the anti-Gly m 5 or Gly m 6 sIgEs are also positive. This then raises the question of which allergens are implicated in a patient's clinical reactions. The DBPCFC (double-blind, placebo-controlled food challenge) is then sometimes the only way to make a reliable diagnosis (15, 19).

We hypothesized that the basophil activation test (BAT) using the soybean molecular allergens Gly m 4, Gly m 5 and Gly m 6, could be useful first to overcome the lack of sensitivity of the soy SPT and of the sIgE assay directed against the overall soy extract and second to highlight the type of soy allergy, according to whether it is linked to storage proteins or to PR-10. The BAT, by its functional aspect, should make it possible to prove *ex vivo* that the degranulation of polynuclear basophils is, according to the patients, specifically induced by stimulation by one type of molecular allergen family, thus providing important additional evidence of the mechanism underlying the allergy. In the case of PR-10-related soy allergies, this might offset the lack of specificity of the anti-Gly m 4 sIgE assay by differentiating, in terms of medical relevance, the patients positive for this assay according to the presence or absence of basophil degranulation in a BAT.

In initial support of our hypothesis, we report here a first confirmation using the basophil activation test of the involvement of PR-10/Gly m 4 in an anaphylactic reaction after ingestion of soymilk in a 27-year-old female patient.

## Original Case Study

A 27-year-old female patient was referred to our center for anaphylaxis rated grade 2 in the Ring and Messmer classification. The reaction occurred in June 2018 during a vacation in Poland, immediately after breakfast. The meal was composed of one apricot and 200 mL of soymilk. Immediately after eating, the patient presented rhinitis, nasal congestion, cough, sneezing, skin rash of the trunk and upper limbs, abdominal pain, and palmoplantar pruritus. There was no laryngeal edema, and no voice modification. Neither pulse nor blood pressure were measured. The patient was not referred to any medical center and no tryptase assay had been performed. The symptoms disappeared spontaneously in a few hours with no treatment.

Detailed medical history revealed spring rhinitis for the previous 3 years, and an oral syndrome on eating raw apple for the previous 2 years. The patient reported episodic consumption of soymilk with no symptoms in 2007–2008, but not liking the taste, never consumed it again until the accident in June 2018. The patient changed her diet in March 2017 to become vegan. Since then, she had been eating soy almost daily, but in cooked forms, almost exclusively as tofu, and occasionally soy sauce after cooking, with no symptoms. The 2018 reaction did not change her eating habits. Since then, besides the previously described forms, she had consumed soy cream in boiled form but stopped because it triggered oral syndromes. She had also tried the lacto-fermented form of soy in cheese substitutes, but again, an oral syndrome quickly appeared. On the other hand, she fully tolerated tempeh (from the fermentation of soybean), but no longer consumed it (not liking the taste).

The patient's family history showed allergic diseases in both parents and one brother.

The diagnosis was confirmed by SPT and determination of sIgEs. Levels of sIgEs for selected allergens were determined using the ImmunoCAP^®^ (Thermo Fisher Scientific^®^) system, using the Phadia 250 equipment according to the manufacturer's instructions (Table 1).

**Table 1:** Results for serum specific IgEs tested.

	**sIgE (kUA/L)**
**Aeroallergens**	
**Birch**	ND
Bet v 1	25.9
Bet v 2	<0.1
**Trophallergens**	
**Soybean**	0.15
Gly m 4	6.41
Gly m5	<0.1
Gly m 6	<0.1
**Apple**	ND
Mal d 1	3.2
Mal d 3	<0.1
**Peach**	ND
Pru p 1	8.24
Pru p 3	<0.1
**Apricot**	0.68

*The sIgEs were tested using the ImmunoCAP method (Thermo Fisher Scientific^®^). The threshold of positivity was set at 0.1 kUA/L*.

*sIgE, specific IgE; ND, not done*.

SPTs were positive for birch extract only. No skin reaction was observed for the other extracts tested (mites, cat, dog, grass, apple, native apple, soy, horse, plantain, herbaceous plants, almond, hazelnut). These negative results thus included both soybean and apple.

Notably, sIgE against whole soybean extract was very weakly positive (0.15 kU/mL). Regarding molecular soy allergens, only anti-Gly m 4 sIgEs were positive, unlike those directed against Gly m 5 and Gly m 6. In addition to anti-Gly m 4 sIgE, elevated sIgEs were found against Bet v 1 and Pru p 1, which also belongs to the protein family of PR-10. Elevated sIgEs were also found against apricot (and against cat and Fel d 1, data not shown).

Based on clinical history, SPT, and sIgEs, we considered the most likely diagnosis to be an anaphylactic reaction to soymilk, mediated by anti-Gly m 4 sIgE and subsequent to an initial birch pollinosis. However, given the negativity of the soy SPT and the quasi-negativity of the sIgE assay directed against the overall soy extract and in order to determine the reactivity threshold, we offered the patient an oral food challenge.

On the patient's refusal to take this test, we sought to confirm the diagnosis using the BAT with soy molecular allergens. We thus performed BATs against soybean extract (Bühlmann, Switzerland), and for the first time to our knowledge against Gly m 4, Gly m 5 and Gly m 6 (Indoor Biotechnologies, USA).

BATs were performed on whole blood using the Flow Cast^®^ and B-CCR^®^ kit (Bühlmann, Switzerland) according to the manufacturer's instructions. Briefly, EDTA whole blood was stimulated in an IL-3 containing buffer for 15 min at 37 °C with increasing concentrations of soybean extract (four concentrations tested in 10-fold dilution ranging from 22.5 to 0.0225 ng/mL for soybean extract, Bühlmann, Switzerland) or its major allergens Gly m 4, Gly m 5 and Gly m 6 (four concentrations ranging from 67.5 to 11.25 ng/mL, Indoor Biotechnologies, USA). Monoclonal antibody recognizing the high-affinity IgE binding receptor (FcεRI) and *N*-formyl-methionyl-leucyl-phenylalanine were used as positive controls. Before erythrocyte lysis, cells were stained with CD63-FITC, CD203c-PEcy5.5 and CCR3-PE. Basophils were gated as SSC-low/CCR3+, and among these, the CD63+ cells were termed activated basophils. Cells were acquired on an LSR II (Becton Dickinson). At least 300 basophils were analyzed using Flowlogic software (version 7.3, Miltenyi Biotec, Germany). Dead cells and doublet cells were excluded by a FSC/SSC gate and an SSC-A/SSC-H gate, respectively. Basophil activation was expressed as the % CD63 positive basophils (% CD63+) or % CD203c positive basophils (% CD203c+) among SSC-low/CCR3+ cells.

CDmax was defined as the maximal activation and corresponds to the maximum proportion of activated basophils (CD63 or CD203c) at any concentration of allergen.

The cut-off value for positive basophil activation in this study was set at >15% CD63 and CD203c basophils.

After stimulation with soybean extract (from 22.5 ng/mL, then with dilutions of 2.25, 0.225, 0.0225 ng/mL), no degranulation of the polynuclear basophils was found using CD63 marker, but a weakly positive activation was highlighted with CD203c marker (Table 2).

**Table 2:** Results of molecular soybean basophil activation test (BAT).

	**Concentration (ng/mL)**	**Case**	
**Allergen**		**CD63 (%)**	**CD203c (%)**
Negative control	-	1.26	3.06
FcεRI	-	70.7	74.3
fMLP	-	37.5	58.3
F14 (total extract)	22.5	2.11	**18.5**
	2.25	0.19	2.72
	0.225	0.58	2.51
	0.0225	3.02	9.06
Gly m 4	67.5	**51.2**	**77.6**
	45	3.38	**15.4**
	22.5	0.96	6.7
	11.25	0.38	6.9
Gly m 5	67.5	0.19	2.33
	45	0.39	2.91
	22.5	0.78	2.73
	11.25	0.4	3.78
Gly m 6	67.5	0.59	2.55
	45	0.78	2.72
	22.5	0.8	2.79
	11.25	0.84	4.63

*CD63 and CD203c results are expressed as a percentage of the maximum basophil activation obtained (or CD max). The threshold of positivity corresponds to a minimum of 15% of activation*.

*Positive values appear in bold*.

As expected, the BAT was very positive to Gly m 4 at 67.5 ng/mL for CD63 and up to 45 ng/mL for CD203c, but negative at all dilutions for Gly m 5 and Gly m 6.

An ImmunoCAP^®^ ISAC^®^ test (Thermo Fisher Scientific^®^) was also performed to obtain a whole sensitization profile and to explore the other PR-10 family allergens (data not shown). The results confirmed sensitizations against all PR-10 present on the biochip (Act d 8, Aln g 1, Api g 1, Ara h 8, Bet v 1, Cor a 1.0101, Cor a 1.0401, Gly m 4, Mal d 1, and Pru p 1).

In view of all these results, supported by those of the original BAT we developed, we established the diagnosis of anaphylactic reaction to soy, mediated by sensitization against PR-10. Subsequently, an immunotherapy against birch was implemented in the hope of treating the pollinosis and possibly the induced PR-10 soy allergy simultaneously (20). Further evaluation of all the results and particularly of the BAT activation threshold will now be necessary.

## Discussion

Initially, this case interested us because it was highly characteristic in some respects. In particular, it again illustrates the finding, first made by Kleine-Tebbe et al. in 2001, that severe oral allergy syndrome (OAS) and anaphylactic symptoms caused by a PR-10-related protein are likely to occur after consumption of a soy product in a patient with birch pollen allergy (14, 21–23).

Although most patients with PFS related to PR-10 have symptoms of moderate intensity, it is important to counsel them about the dangers of particular highly concentrated food forms such as dietary supplements, fresh fruit juices, smoothies and plant milks, like soymilk in our example (24). This seems to hold particularly for soybeans, probably due in part to a greater resistance of Gly m 4 to heat or gastric digestion than other PR-10 (15, 25).

This case is also interesting because it further illustrates that vegan diets may play a role in the development of food anaphylaxis (26). Our patient became vegan in 2017, and then started consuming much more soy, reacting for the first time to it in 2018. We can therefore legitimately suspect that the change in the patient's dietary habits may have played a role in the advent of the allergy.

Another characteristic point is the negativity of the soy SPT and the near negativity of the sIgE assay against total soy extract. This illustrates the weakness of the diagnostic tools classically at our disposal to diagnose soybean allergy when it is a form mediated by sIgE directed against PR-10.

In this example, the anti-Gly m 4 sIgE assay was already informative (27). However, when an oral food challenge cannot be performed, and since a very significant proportion of patients presenting birch pollinosis have anti-Gly m 4 sIgE without having a PR-10-mediated soy allergy, it is useful to have another confirmatory test for etiological purposes (15).

By demonstrating *ex vivo* the degranulation of basophilic polynuclear cells in contact with Gly m 4, the original BAT that we developed provides valuable mechanistic evidence that the anti-Gly m 4 sIgEs previously measured by the serum unitary assays have a functional activity and clinical relevance. The BAT mimics *ex vivo* what must have happened *in vivo* in the patient during the soy anaphylactic reaction. The BAT to soybean molecular allergens thus provides important evidence for the medical relevance of the sensitization measured against Gly m 4.

In our case, it is striking to see how closely the results of the sIgEs and BAT assays agreed. Thus, the sIgE assay directed against the overall extract was very weakly positive, as was that of the BAT against this same extract (CD63 negative and CD203c just above the threshold). Likewise, the anti-Gly m 4 sIgEs were quite high when the BAT against this allergen was very sharply positive for both CD63 and CD203c. Conversely, the anti-Gly m 5 and Gly m 6 sIgEs were fully negative, as was the BAT using these two allergens. This excellent agreement strengthens the relevance of the results of our original BAT based on soy molecular allergens.

Two very recent articles have focused on the use of BATs in the diagnosis of soy allergy. However, in both cases, the authors used only a total soy extract (soymilk proteins for one and natto extracts and soybean extract for the other, respectively) (28, 29). Although they both concluded that the BAT was of interest in this indication, their test method cannot distinguish between the two types of soy allergy at a molecular level and so does not seem to us to be able to answer all the questions raised by the diagnosis of soy allergy.

To conclude, in view of our preliminary results, we consider that in addition to clinical history, SPT and sIgE assays, BATs using soybean molecular allergens can be of use in the diagnosis of soy allergy in the near future. We have recently started to use BATs in practice, and will soon be working to define their place in the diagnostic tree, in particular in relation to oral food challenges, which they might even obviate. By following the basophil activation threshold over time, we can also envisage a place for BATs in the follow-up of possible oral soy immunotherapies, or in that of birch immunotherapies, in order to detect a possible concomitant induced effect on the allergy associated with PR-10 soy Gly m 4. These possibilities will be the subject of future studies.

## Data Availability Statement

The original contributions presented in the study are included in the article/supplementary material, further inquiries can be directed to the corresponding author.

## Ethics Statement

Ethical review and approval was not required for the study on human participants in accordance with the local legislation and institutional requirements. The patient/participant provided their written informed consent to participate in this study.

## Author Contributions

BE designed the study and wrote the first draft of manuscript. BB and JC conducted the experiments. BE, JC, and BB analyzed the data and had access to and verified all underlying data. BB made the tables. All authors reviewed the manuscript and gave significant input. The final version of this paper was reviewed and approved by all authors.

## Conflict of Interest

The authors declare that the research was conducted in the absence of any commercial or financial relationships that could be construed as a potential conflict of interest.

## Publisher's Note

All claims expressed in this article are solely those of the authors and do not necessarily represent those of their affiliated organizations, or those of the publisher, the editors and the reviewers. Any product that may be evaluated in this article, or claim that may be made by its manufacturer, is not guaranteed or endorsed by the publisher.
